# Supplementary material for: Corticosterone-Mediated Physiological Stress Alters Liver, Kidney, and Breast Muscle Metabolomic Profiles in Chickens
Source: Animals (Basel). 2021 Oct 26;11(11):3056. doi: 10.3390/ani11113056 (PMC8614290; doi:10.3390/ani11113056)
Supplement: Supplementary file 1 [file animals-11-03056-s001.zip › animals-1383565-supplementary.pdf]

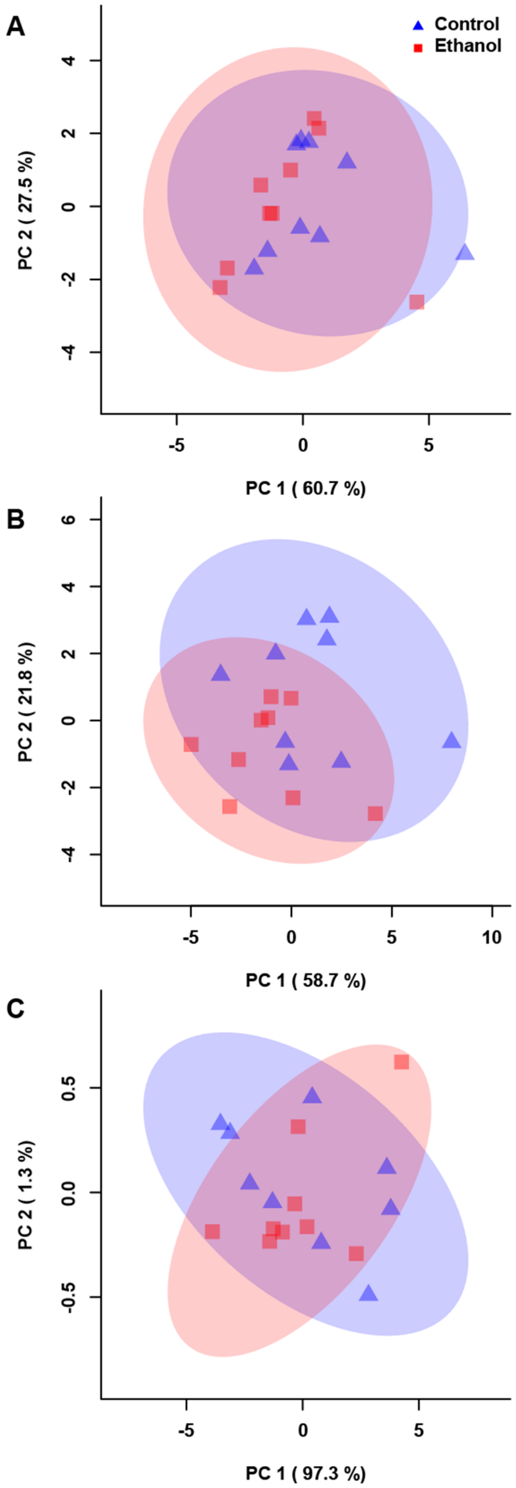

Figure S1. Principal component analysis scores plots for the control treatment vs the ethanol treatment. (A) Kidney. (B) Liver. (C) Breast muscle. Each triangle or square represents one chicken (n=9), plotted using a list of bins found to be statistically significant via paired T-test and/or multivariate variable importance analysis based on random variable combination analysis. The X and Y axes show principal components with brackets indicating percent variance and the shaded ellipse representing the 95% confidence interval.

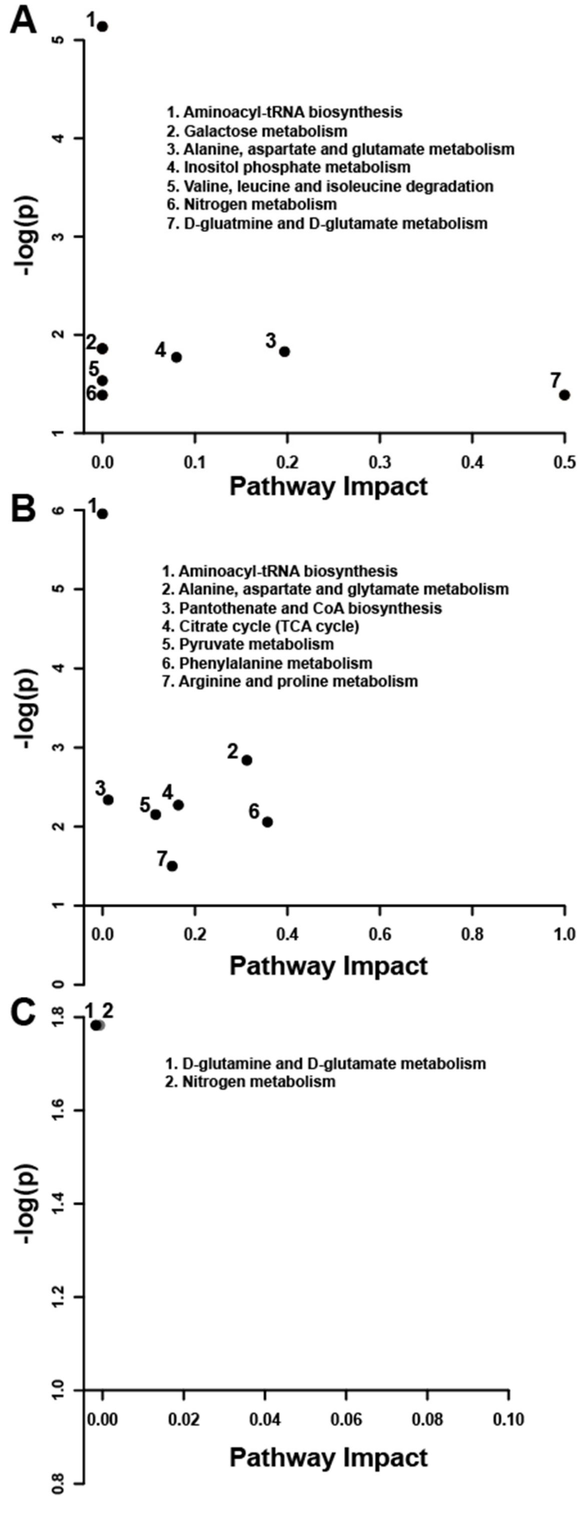

Figure S2. Metabolomic pathway analysis showing all matched pathways according to p-values from pathway enrichment analysis and pathway impact values for the control treatment vs the ethanol treatment. (A) Kidney. (B) Liver. (C) Breast muscle. A larger value on the y-axis indicates a lower *P*-value. The x-axis gives the pathway impact. Only metabolic pathways with $P \leq 0.050$ are labeled. This figure was created using the lists of metabolites identified as significant by paired T-test and/or multivariate variable importance analysis based on random variable combination analysis.

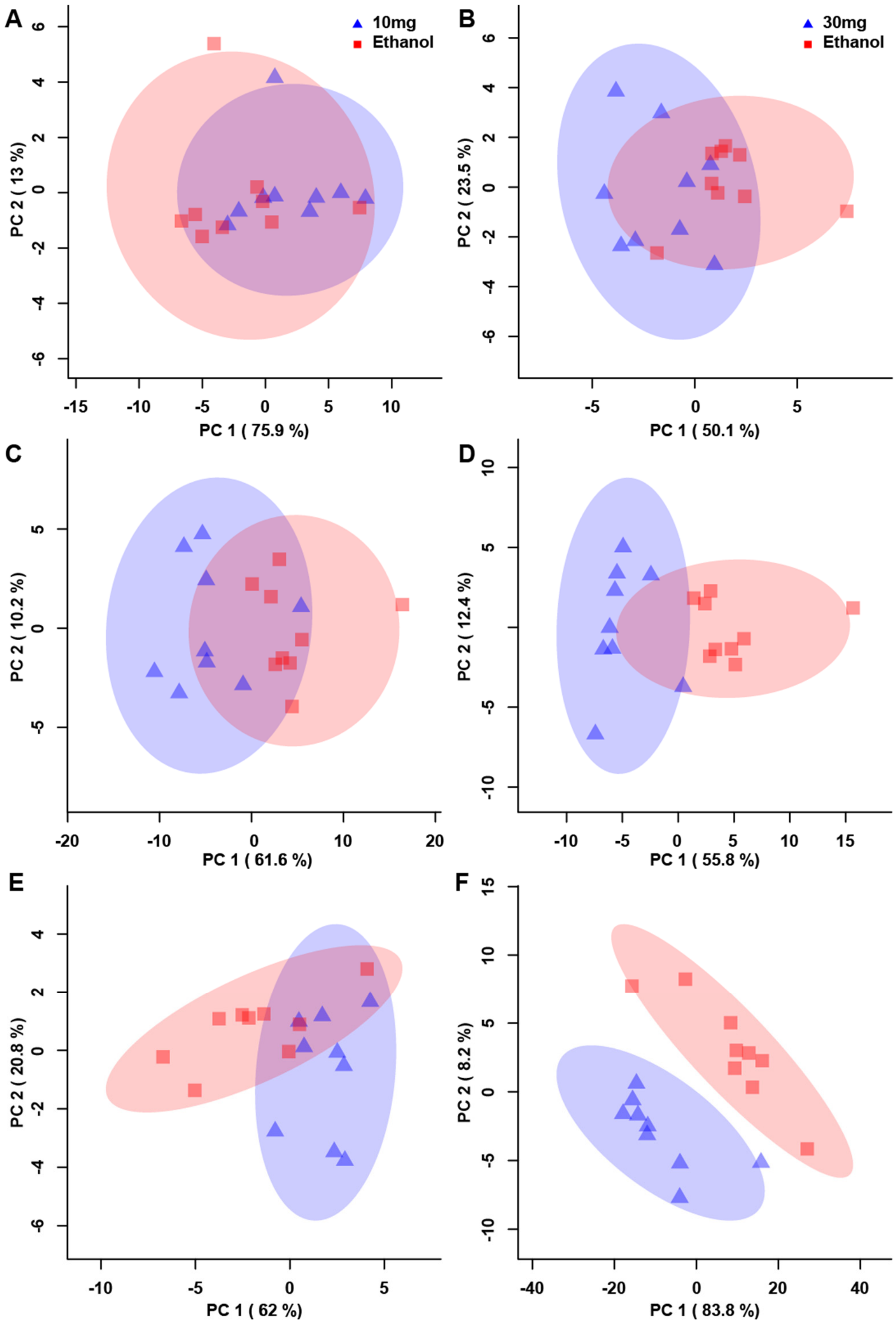

Figure S3. Principal component analysis scores plots for the corticosterone treatments vs the ethanol control treatment. (A) Kidney – 10 mg L^{-1} corticosterone. (B) Kidney – 30 mg L^{-1} corticosterone. (C) Liver – 10 mg L^{-1} corticosterone. (D) Liver – 30 mg L^{-1} corticosterone. (E) Breast muscle – 10 mg L^{-1} corticosterone. (F) Breast muscle – 30 mg L^{-1} corticosterone. Each triangle or square represents one chicken under study (n=9), plotted using a list of bins found to be statistically significant via paired T-test and/or multivariate variable importance analysis based on random variable combination analysis. The X and Y axes show principal components with brackets indicating percent variance and the shaded ellipse representing the 95% confidence interval.

Table S1. Number of significantly altered bins across all tissues and corticosterone (**CORT**) doses.

**Kidney**	**Paired T-test**	**VIAVC**	**Common to both tests**
10 mg L^{-1} CORT	63	12	4
30 mg L^{-1} CORT	45	7	1
**Liver**			
10 mg L^{-1} CORT	236	5	3
30 mg L^{-1} CORT	217	3	3
**Breast**			
10 mg L^{-1} CORT	31	10	1
30 mg L^{-1} CORT	251	75	72

Table S2. *P*-values of metabolites found to be significant altered in kidneys of birds administered 10 mg L^{-1} corticosterone (**CORT**) and 30 mg L^{-1} CORT as compared to ethanol alone as determined by a paired T-test and/or the variable importance analysis based on random variable combination analysis. Metabolite regulation is shown as a percentage of the difference between the CORT treatment vs ethanol treatment. Metabolites for which more than one nuclear magnetic resonance peak was identified as significant are represented as metabolite 1, metabolite 2, … metabolite n.

Treatment	Metabolite	Chemical shift (ppm)	T-test *P*-value	VIAVC *P*-value	Regulation
10 mg L^{-1} CORT	S-Adenosylhomocysteine 1	4.45	0.0019		13.90
	Fumaric Acid	6.53	0.002	4.450E-60	23.13
	Nicotinate/Nicotinic acid	8.27	0.0039		28.74
	Phenylalanine 1	3.11	0.0057		10.30
	sn-Glycero-3-phosphocholine 1	3.88	0.0073		12.84
	Proline 1	3.36	0.0078		16.50
	Lactate 1	1.32	0.0078		-31.41
	Malate 1	4.30	0.0115		11.85
	Glutamate 1	2.15	0.0117		-15.04
	Glutamate 2	2.11	0.0117		-7.59
	Lactate 2	1.35	0.0117		-23.43
	Malate 2	4.30	0.0129		21.82
	N-Acetylaspartate 1	4.38	0.0137		20.79
	UNIDENTIFIED 1	3.87	0.015		11.11
	AMP 1	6.15	0.0178		38.70
	3-Methyladenine 1 and S-Adenosylhomocysteine 2	8.28	0.0193		22.84
	UDP-N-Acetylglucosamine	6.00	0.0195		19.19
	Lactate 3	4.13	0.0195		-20.31
	Lactate 4	4.12	0.0195		-18.84
	Glutamate 3	2.12	0.0195		-9.05
	Isoleucine 1	0.96	0.0195		-10.19
	Isoleucine 2	0.95	0.0195		-11.45
	sn-Glycero-3-phosphocholine 2	3.70	0.0213		16.11
	Inosine 1	8.37	0.022	2.055E-07	45.32
	N6-Acetyl-L-lysine	3.20	0.0228		15.06
	N-Acetylaspartate 2	4.39	0.0248		14.45
	O-Phosphocholine	3.59	0.0254		7.67
	1,3-Dimethyluric Acid	3.45	0.0254		16.00
	Citicoline 1	4.29	0.0255		20.24
	Glucose 1	3.84	0.0269		18.88
	Choline 1	4.07	0.0273		13.16
	4-Hydroxyproline 1	3.48	0.0281		13.43
	Xylulose	4.37	0.0282		21.54
	AMP 2 and Carnosine 1	4.50	0.0287		26.57

	S-Adenosylhomocysteine 3	2.99	0.0305		8.48
	Glutamate 4	2.06	0.0309		-6.13
	AMP 3 and Carnosine 2	4.51	0.0311		29.46
	S-Adenosylhomocysteine 4	8.38	0.0314		41.10
	UMP and Citicoline 2	4.42	0.0322		20.47
	Proline 2 and 1,5-Anhydrosorbitol 1	3.36	0.0323		11.98
	UNIDENTIFIED 2	4.24	0.0325		9.62
	Uridine 1	4.22	0.034		13.19
	sn-Glycero-3-phosphocholine 3	3.69	0.0347		14.16
	sn-Glycero-3-phosphocholine 4	3.89	0.0348		13.36
	(R)-3-Hydroxybutyric acid	1.21	0.0351		16.83
	AMP 4	8.60	0.0354	1.494E-16	31.76
	Glutamate 5	2.13	0.0354		-10.01
	Uridine 2	4.23	0.0362		19.41
	Agmatine and Carnosine 3	3.07	0.039		7.09
	Glutamate 6	2.34	0.0391		-11.71
	Glutamate 7	2.07	0.0391		-8.36
	Proline 3 and Glucose 2	3.39	0.0394		14.34
	Choline 2	4.06	0.0401		18.50
	Citicoline 3	4.28	0.0408		18.04
	Citicoline 4	4.33	0.0411		14.94
	3-Mercaptopyruvic acid	2.87	0.0413		21.55
	3-Methyladenine 2	3.98	0.0424		9.33
	1,5-Anhydrosorbitol 2	3.44	0.0438		8.65
	Inosine 2	6.10	0.0443	7.267E-09	45.66
	Phenylalanine 2	3.12	0.0457		4.85
	Inosinic Acid	8.57	0.047		44.15
	Pyruvate	2.39	0.047		10.26
	N-Acetylaspartate 3 and Citicoline 5	4.41	0.0492		27.92
	Malate 3	2.66	0.0514	7.272E-47	12.97
	Inosine 3	6.11	0.0568	1.078E-19	51.44
	Malate 4	2.38	0.0615	1.668E-44	6.35
	UNIDENTIFIED 3	7.57	0.2198	1.749E-04	31.36
	N-Methylhydantoin	2.93	0.3445	6.001E-34	10.16
	Aspartate	2.81	0.4076	1.320E-23	4.55
	4-Hydroxyproline 2 and Isovalerylglycine	2.17	0.5372	1.172E-42	2.20
	Carnitine	2.45	0.6014	5.047E-38	-3.14
30 mg L^{-1} CORT	Acetic acid	1.93	0.0004		-23.13
	4-Hydroxyproline 1	3.48	0.0009		39.50
	UNIDENTIFIED 1	1.78	0.0018		-18.88
	Glutamate 1	2.13	0.0033		-20.46

	1,5-Anhydrosorbitol	3.44	0.0036		33.31
	Fumaric Acid	6.53	0.0036		29.42
	Glucose-6-phosphate	3.99	0.0039		-19.09
	Glutamate 2	2.15	0.0039		-32.83
	Glucose 1	3.84	0.0052		34.01
	L-Lysine	3.75	0.0053		15.20
	UNIDENTIFIED 2	4.00	0.007		-20.85
	Choline 1	3.53	0.0078		17.54
	Glutamate 3	2.33	0.0116		-14.21
	Glutamate 4	2.12	0.0117		-17.53
	UNIDENTIFIED 3	3.98	0.0117		-14.13
	UNIDENTIFIED 4	0.97	0.0117		-18.44
	Glutamate 5	2.06	0.0118		-13.58
	Choline 2	3.52	0.0195		29.51
	Glutamate 6	2.11	0.0195		-13.66
	Phosphorylcholine 1	4.17	0.0207		-19.51
	Glucose 2	3.71	0.0229		24.33
	UNIDENTIFIED 5	3.27	0.023		-35.03
	Serine	4.01	0.0246		-15.08
	UNIDENTIFIED 6	2.71	0.026		-15.43
	Isoleucine 1	0.95	0.0273		-25.52
	Malate	4.31	0.0273		12.99
	Dimethylamine and Sarcosine	2.74	0.0291		-10.96
	Beta-Alanine	3.18	0.0308		-12.60
	Glutamate 7	2.05	0.032		-17.57
	sn-Glycero-3-phosphocholine 2 and Isoleucine 2	3.69	0.0339		7.90
	Argininosuccinic acid	1.92	0.0391		-7.81
	Glycerol 1	3.67	0.0391		9.35
	Glycine	3.57	0.0391		-25.30
	Isoleucine 3	0.94	0.0391		-16.92
	Pyroglutamic Acid 1	2.42	0.0391	4.908E-15	-24.88
	sn-Glycero-3-phosphocholine 1	3.69	0.0391		20.40
	Glucose 3	3.49	0.0412		22.87
	Creatine	3.94	0.0422		-11.89
	D-Glucuronic acid	3.75	0.0423		18.74
	Malate	4.30	0.0443		15.87
	Creatinine and Creatine Phosphate	3.04	0.0445		-23.67
	Phosphorylcholine 2	3.60	0.0471		-10.37
	Glycerol 2	3.65	0.0491		8.66
	Choline 3	4.07	0.0663	2.548E-09	21.64
	Myoinositol	3.28	0.0813	1.064E-21	-19.12
	UNIDENTIFIED 7	4.20	0.7344	4.809E-10	0.31

	N-Methylhydantoin	2.93	0.748	1.860E-17	10.99
	Pyroglutamic Acid 2 and 3-Phenylpropionate	2.49	0.8203	2.616E-21	-2.85
	UNIDENTIFIED 8	2.43	0.8867	4.816E-13	-1.03
	4-Hydroxyproline 2 and Isovalerylglycine	2.17	0.9102	1.221E-11	-5.71

Table S3. *P*-values of metabolites found to be significant altered in livers of birds administered 10 mg L^{-1} corticosterone (**CORT**) and 30 mg L^{-1} CORT as compared to ethanol alone as determined by a paired T-test and/or the variable importance analysis based on random variable combination analysis. Metabolite regulation is shown as a percentage of the difference between the CORT treatment vs ethanol treatment. Metabolites for which more than one nuclear magnetic resonance peak was identified as significant are represented as metabolite 1, metabolite 2, … metabolite n.

**Treatment**	**Metabolite**	**Chemical shift (ppm)**	**T-test *P*-value**	**VIAVC *P*-value**	**Regulation**
10 mg L^{-1} CORT	UNIDENTIFIED 1	8.02	0.0004		-36.78
	UNIDENTIFIED 2	3.17	0.0005		-24.67
	Mannose	5.20	0.0011		46.89
	4-Hydroxyproline 1	3.37	0.0019	0.00166	-19.53
	Inosine 1	4.45	0.002		-24.91
	Tryptophan 1	7.55	0.0021		-36.39
	Coenzyme A	0.76	0.0028		-27.10
	UNIDENTIFIED 3	4.46	0.003		-27.16
	UMP 1	4.42	0.0031		-26.20
	Phenylalanine 1	7.35	0.0033		-37.46
	Quinone	6.81	0.0036		-41.61
	Tyrosine 1	6.92	0.0037		-43.42
	UNIDENTIFIED 4	4.41	0.0037		-25.20
	Inosine 2	4.45	0.0038		-20.24
	UNIDENTIFIED 5	0.87	0.0038		-32.91
	(R)-3-Hydroxybutyric Acid 1	1.22	0.0039		-45.42
	Glycerophosphocholine 1	3.69	0.0039		28.82
	N-Carbamoylaspartate 1	2.47	0.0039		-35.46
	N-Methylhydantoin	4.10	0.0039		-22.30
	UNIDENTIFIED 6	2.48	0.0039		-32.42
	UNIDENTIFIED 7	2.32	0.0039		-27.16
	UNIDENTIFIED 8	1.19	0.0039		-26.02
	Valine 1	2.30	0.0039		-33.74
	L-Threonine 1	4.28	0.0046		-23.04
	Tryptophan 2	7.21	0.0046		-34.30
	Myoinositol 1	3.63	0.0047		-31.65
	Phenylalanine 2	7.33	0.0049		-35.37
	Valine 2	2.29	0.0051		-27.60
	O-Phosphocholine 1	3.59	0.0054		17.05
	Phenylalanine 3	7.45	0.0055		-31.08
	Tyrosine 2	7.19	0.0055		-36.15
	Tryptophan 3	7.75	0.0057		-29.20
	Myoinositol 2	4.08	0.0062		-37.47
	Gamma-Glutamylcysteine 1	2.19	0.0063		-16.40
	Tyrosine 3	7.20	0.0065		-37.41

	UNIDENTIFIED 9	3.00	0.0065		-16.43
	Uridine 1	7.88	0.0071		-35.83
	UNIDENTIFIED 10	4.46	0.0072		-26.05
	Isoleucine 1	0.94	0.0073		-28.97
	Tryptophan 4	7.29	0.0073		-34.32
	Isoleucine 2	0.93	0.0074		-29.89
	Beta-Alanine 1	3.17	0.0075		-24.23
	Gamma-Glutamylcysteine 2	2.58	0.0075		-14.88
	Flavin Mononucleotide	7.81	0.0076		-36.70
	(R)-3-Hydroxybutyric Acid 2	2.42	0.0078		-26.88
	(R)-3-Hydroxybutyric Acid 3	1.20	0.0078		-52.53
	Glycerophosphocholine 2	3.69	0.0078		28.34
	Isoleucine 3	1.26	0.0078		-20.34
	N-Acetylglutamate 1	2.23	0.0078		-20.19
	Phenylalanine 4	3.16	0.0078		-19.48
	Tyrosine 4	3.08	0.0078		-18.98
	Tyrosine 5	3.07	0.0078		-20.91
	UNIDENTIFIED 11	4.49	0.0078		-23.03
	UNIDENTIFIED 12	2.32	0.0078		-22.57
	UNIDENTIFIED 13	2.31	0.0078		-28.06
	Proline 1	3.34	0.008		18.45
	Gluconate	4.13	0.0081		21.36
	Phenylalanine 5	7.44	0.0083		-32.78
	Phenylalanine 6	7.37	0.0083		-28.40
	4-Hydroxyproline 2	2.16	0.0086		-17.81
	Inosine 3	4.44	0.0087		-21.19
	Allantoin	5.40	0.0089		-30.92
	UNIDENTIFIED 14	0.95	0.009		-30.19
	Glutamate 1	2.11	0.0091		-25.80
	UMP 2	4.43	0.0092		-24.52
	1-Methyladenosine	6.09	0.0094		-14.62
	UNIDENTIFIED 15	4.25	0.0094		-22.29
	Norspermidine 1	1.69	0.0095		-27.66
	O-Phosphocholine 2	3.24	0.0095		27.37
	Xanthine 1	7.93	0.0095		-30.11
	Gamma-Glutamylcysteine 3	2.17	0.0097		-16.27
	Myoinositol 3	3.54	0.0098		-8.45
	Glucose 1	3.42	0.01		11.83
	Phenylalanine 7	7.38	0.0101		-30.68
	UNIDENTIFIED 16	3.36	0.0101		12.00
	Myoinositol 4	3.64	0.0104		-22.60
	Tyrosine 6	6.91	0.0104		-33.50
	UNIDENTIFIED 17	4.40	0.0105		-19.78

	UNIDENTIFIED 18	2.15	0.0107		-22.45
	UNIDENTIFIED 19	5.99	0.0109		18.45
	Valine 3	2.28	0.0113		-26.72
	Pantothenic acid 1	0.92	0.0114		-22.00
	Inosine 4	6.08	0.0115		-22.16
	Glucose 2	3.89	0.0117		12.65
	L-Threonine 2	4.27	0.0117		-20.98
	Niacinamide 1	7.60	0.0117		-19.54
	Tyrosine 7	3.09	0.0117		-16.70
	UNIDENTIFIED 20	4.47	0.0117		-25.14
	Uridine 2	7.89	0.0117		-34.37
	Uridine 3	5.93	0.0117		-37.72
	Uridine 4	5.92	0.0117		-37.22
	Xanthine 2	7.91	0.0118		-18.70
	Tryptophan 5	7.73	0.0121		-32.14
	Myoinositol 10	3.30	0.0122		-29.32
	L-Threonine 3	4.25	0.013		-20.82
	Betaine and Trimethylamine N-Oxide	3.27	0.0131		-32.81
	UNIDENTIFIED 21	0.91	0.0136		-27.43
	Ribose	5.40	0.0138		-22.85
	UNIDENTIFIED 22	7.57	0.0139		-23.37
	UNIDENTIFIED 23	0.98	0.0139		-27.71
	Myoinositol 5	3.56	0.0141		-9.29
	UNIDENTIFIED 24	7.92	0.0143		-18.63
	UNIDENTIFIED 25	1.70	0.0143		-27.47
	Norspermidine 2	1.68	0.0145		-22.91
	Gamma-Glutamylcysteine 4	2.59	0.0146		-15.30
	Methionine	2.14	0.0146		-28.84
	Glucose 3	3.43	0.0147		12.70
	Citric Acid 1	2.53	0.0149		-15.65
	Glutamate 2	2.07	0.0149		-18.61
	Beta-Alanine 2 and Isocitrate 1 and Argininosuccinic acid 1	2.56	0.015		-12.19
	Uridine 5	5.91	0.015		-31.84
	Valine 4	2.28	0.015		-26.66
	Myoinositol 6	3.56	0.0152		-9.56
	Proline 2	3.35	0.0152		17.50
	Myoinositol 7	3.31	0.0153		-27.01
	Gamma-Glutamylcysteine 5	2.99	0.0154		-12.54
	UNIDENTIFIED 26	2.49	0.0154		-18.18
	Myoinositol 8	3.61	0.0159		-22.07
	UNIDENTIFIED 27	1.42	0.0161		-21.41
	(R)-3-Hydroxybutyric Acid 4	2.43	0.0163		-28.10

	UNIDENTIFIED 28	0.96	0.0163		-29.47
	Glucose 4	3.39	0.0164		11.93
	UNIDENTIFIED 29	4.57	0.0164		-30.89
	1-Methyladenine	8.31	0.0166		-15.59
	Dimethylamine	2.50	0.017		-16.02
	Pantothenic acid 2	0.89	0.0176		-25.76
	Phenylalanine 8	7.42	0.0177		-26.17
	Phenylalanine 9	7.40	0.0178		-25.88
	7-Methyladenine 1	8.11	0.018		-24.15
	Inosine triphosphate	8.50	0.0181		-18.01
	N-Carbamoylaspartate 2	2.46	0.0181		-37.36
	UNIDENTIFIED 30	1.64	0.0182		-17.96
	UDP-N-Acetylglucosamine 1	5.98	0.0186		18.87
	Valine 5	2.27	0.0186		-24.88
	Valine 6	2.27	0.0191		-24.55
	Gamma-Glutamylcysteine 6	2.22	0.0195		-16.39
	Gamma-Glutamylcysteine 7	2.21	0.0195		-15.16
	Gamma-Glutamylcysteine 8	2.20	0.0195		-15.18
	Glucose 5	3.71	0.0195	0.00312	17.22
	Isoleucine 4	1.26	0.0195		-15.96
	N-Acetylglutamate 2	2.24	0.0195		-19.70
	Niacinamide 2	7.62	0.0195		-20.02
	Nicotinurate 1	7.61	0.0195		-16.33
	Tryptophan 6	7.28	0.0195		-28.97
	Tryptophan 7	7.30	0.0195		-30.56
	Tyrosine 8	3.06	0.0195		-11.85
	UDP-N-Acetylglucosamine 2	5.98	0.0195		22.09
	UNIDENTIFIED 31	4.56	0.0195		-28.46
	UNIDENTIFIED 32	4.49	0.0195		-20.11
	UNIDENTIFIED 33	4.48	0.0195		-22.41
	UNIDENTIFIED 34	1.18	0.0195		-18.12
	Isoleucine 5	1.02	0.0196		-26.51
	Fucose	4.55	0.0203		-21.26
	Citric Acid 2	2.57	0.0204		-12.16
	Argininosuccinic acid 2	2.54	0.0205		-12.79
	Selenomethionine 1	2.64	0.0207		-26.55
	Adenosine Monophosphate 1	8.60	0.021	0.00275	41.94
	Proline 3	3.35	0.0211		19.15
	UNIDENTIFIED 35	4.14	0.0217		15.45
	Adenosine Monophosphate 2 and Cytidine Monophosphate 1	6.15	0.0221		34.84
	Selenomethionine 2	2.65	0.0226		-26.70
	Nicotinurate 2	8.72	0.0234		-14.76

	L-Threonine 4	4.26	0.0238		-18.65
	Norspermidine 3	1.67	0.024		-18.87
	Glycerophosphocholine 3	3.68	0.0247		18.22
	UNIDENTIFIED 36	1.63	0.0261		-15.65
	UNIDENTIFIED 37	1.15	0.0261		-18.95
	Adenine	8.22	0.0264		-23.43
	UMP 3	8.09	0.0264		-49.31
	Tryptophan 8	7.56	0.0265		-23.45
	UNIDENTIFIED 38	1.43	0.0266		-19.37
	Thiamine pyrophosphate	8.05	0.027		-16.90
	Fructose	4.03	0.0273		15.22
	Glucose 10	3.53	0.0273		6.84
	Glucose 11	3.45	0.0273		13.62
	Glucose 12	3.80	0.0273		8.44
	Glucose 6	5.24	0.0273		21.35
	Glucose 7	3.86	0.0273		10.57
	Glucose 8	3.84	0.0273		13.22
	Glucose 9	3.83	0.0273		12.71
	Nicotinurate 3	8.95	0.0273		-15.33
	Nicotinurate 4	8.71	0.0273		-14.90
	S-Adenosylhomocysteine 1	8.39	0.0273		-26.52
	Uridine 6	4.36	0.0273		-25.18
	Valine 7	2.26	0.0275		-21.66
	Valine 8	0.99	0.0282		-23.44
	Inosine 5 and Hypoxanthine	8.23	0.0302		-23.67
	Uridine 7	5.94	0.0304		-29.41
	Argininosuccinic acid 3 and Isocitrate 2	2.52	0.0312		-12.97
	S-Adenosylhomocysteine 1 and Inosine 5 and Adenosine	8.36	0.0323		-9.50
	Isocitrate 3	2.51	0.0327		-13.26
	Myoinositol 9	3.55	0.0336		-8.70
	Methionine Sulfoxide	2.77	0.034		-20.13
	UNIDENTIFIED 39	1.66	0.0347		-17.11
	7-Methyladenine 2	8.19	0.0358		-13.83
	Adenosine Monophosphate 3 and Cytidine Monophosphate 2	6.14	0.0365		29.75
	Isoleucine 6	1.01	0.0366		-24.80
	Valine 9	1.04	0.037		-23.31
	Imidazole	8.32	0.0373		6.19
	UNIDENTIFIED 40	3.85	0.0374		10.16
	Glucose 13	3.78	0.0385		3.73
	Glutamate 3	2.13	0.0389		-21.32
	(R)-3-Hydroxybutyric Acid 5	2.39	0.0391		-22.09
	Beta-Alanine 3	3.18	0.0391		-16.47

	Glucose 14	3.84	0.0391		14.28
	Glucose 15	3.76	0.0391		11.64
	Glucose 16	3.73	0.0391		13.64
	Glucose 17	3.72	0.0391		15.32
	Glycogen	5.41	0.0391		45.07
	L-Threonine 5	4.25	0.0391		-17.25
	N-Acetylglutamate 3	2.25	0.0391		-19.22
	Nicotinurate 5	8.73	0.0391		-15.74
	Nicotinurate 6	7.62	0.0391		-15.76
	Phenylalanine 10	3.15	0.0391		-20.66
	Phenylalanine 11	3.14	0.0391		-17.66
	Tyrosine 9 and Beta-Alanine 4	3.19	0.0391		-16.04
	UNIDENTIFIED 41	4.52	0.0391		-16.81
	Uridine 8	4.24	0.0391		-21.65
	Uridine 9	4.23	0.0391		-18.24
	UNIDENTIFIED 42	0.90	0.0394		-22.91
	Pi-Methylhistidine	7.16	0.0397		-24.13
	UNIDENTIFIED 43	2.42	0.0422		-25.33
	Methylmalonate 1	1.24	0.0427		-16.16
	Methylmalonate 2	1.23	0.0432		-15.63
	UDP-N-Acetylglucosamine 3	7.95	0.0434		8.70
	UNIDENTIFIED 44	3.62	0.0437		-13.62
	Valine 10	1.05	0.045		-23.13
	UNIDENTIFIED 45	1.65	0.0451		-16.78
	Anserine	7.15	0.0454		-20.47
	Gamma-Glutamylcysteine 9	2.98	0.0461		-10.03
	Glutamate 4	2.10	0.0472		-16.12
	UNIDENTIFIED 46	1.65	0.0481		-15.62
	Glycerol	3.81	0.0488		5.69
	3-Methyladenine	8.29	0.0492		20.31
	Thiamine	8.06	0.0493		-17.94
	3-Mercaptopyruvic Acid	2.86	0.0547	0.00208	11.19
	Threonate	4.02	0.2451	0.00039	6.81
30 mg L^{-1} CORT	Betaine and Trimethylamine N-Oxide	3.274811	0.00001	1.40E-03	-55.02
	Coenzyme A	0.762045	0.00001		-43.08
	Glycerophosphocholine 1	3.69339025	0.00001		48.05
	Mannose	5.19833	0.00001		75.00
	UNIDENTIFIED 1	3.8724865	0.00001	9.85E-04	26.44
	UNIDENTIFIED 2	3.6352585	0.00001		21.92
	UNIDENTIFIED 3	3.16603875	0.00001		-36.08
	Glycerophosphocholine 2 and Maltose	3.67813075	0.0001		39.49
	Glycerophosphocholine 3	3.68501725	0.0002	7.60E-04	50.20
	Quinone	6.81344075	0.0002		-55.24

	1-Methylguanosine	8.01996375	0.0003		-35.27
	Glycerophosphocholine 4	3.2250335	0.0004		42.15
	Inosine 1	4.451543	0.0004		-27.68
	Tryptophan 1	7.55075	0.0004		-38.80
	O-Phosphocholine 1	3.5931865	0.0005		31.26
	Nicotinurate 1	8.253198	0.0008		-22.60
	Tryptophan 2	7.733	0.0009		-26.08
	Allantoin 1	5.3965	0.0011		-33.81
	Tryptophan 3	7.28869675	0.0012		-32.83
	Tryptophan 4	7.214888	0.0012		-33.75
	Valine 1	2.289244	0.0012		-27.33
	Phenylalanine 1	7.447597	0.0013		-36.26
	Nicotinurate 2	8.7221	0.0015		-26.86
	Tyrosine 1	7.1892	0.0015		-36.82
	Myoinositol 1	4.0815	0.0016		-34.35
	O-Phosphocholine 2	3.236193	0.0016		45.15
	4-Hydroxyproline	3.3730335	0.0019		-25.92
	UNIDENTIFIED 4	4.248287	0.002		-21.32
	Beta-Alanine 1	3.17114475	0.0022		-30.05
	UNIDENTIFIED 5	3.95	0.0022		26.25
	Glucose 1	3.7765	0.0023		4.89
	UNIDENTIFIED 6	3.848027	0.0023		10.55
	Gluconate	4.127	0.0024		29.28
	Tyrosine 2	7.204088	0.0024		-35.70
	Valine 2	2.2769985	0.0024		-24.99
	Isoleucine 1	0.9422585	0.0027		-33.68
	Histamine 1	7.93006425	0.0028		-44.51
	Inosine 2	4.4453885	0.0028		-22.06
	Inosine 3	6.08357225	0.0029		-31.71
	Tryptophan 5	7.277904	0.003		-27.31
	7-Methyladenine 1	8.1092455	0.0031		-26.10
	Inosine triphosphate	8.5015	0.0031		-24.98
	Myoinositol 2	3.6284505	0.0031		-24.92
	Myoinositol 3	3.312	0.0032		-24.39
	Tryptophan 6	7.56175	0.0033		-27.83
	UDP-N-Acetylglucosamine 1	5.97683275	0.0034		41.80
	Valine 3	2.281673	0.0034		-25.11
	Tyrosine 3	6.9070865	0.0038		-36.27
	(R)-3-Hydroxybutyric Acid 1	1.2171995	0.0039		-35.05
	Glucose 2	3.8908375	0.0039		11.25
	Glucose 3	3.85905	0.0039		12.93
	Glucose 4	3.830115	0.0039		11.62
	Glucose 5	3.70511225	0.0039		15.95

	Glucose 6 and Glycerophosphocholine 5	3.2476595	0.0039		20.84
	Glycogen 1	5.436698	0.0039		41.55
	Glycogen 2	5.4231095	0.0039		58.92
	Glycogen 3	5.4149115	0.0039		51.66
	NADP+	9.303	0.0039		40.69
	Nicotinurate 3	8.9535	0.0039		-23.92
	Nicotinurate 4	8.7258	0.0039		-27.84
	Nicotinurate 5	8.71475	0.0039		-25.78
	Nicotinurate 6	7.624	0.0039		-24.39
	Nicotinurate 7	7.615	0.0039		-25.20
	Nicotinurate 8	7.609	0.0039		-25.09
	Nicotinurate 9	7.601	0.0039		-27.14
	Phenylalanine 2	7.43567725	0.0039		-42.46
	Phenylalanine 3	7.3454015	0.0039		-49.18
	Phenylalanine 4	7.3313645	0.0039		-49.59
	Phenylalanine 5	3.1581545	0.0039		-26.59
	Phenylalanine 6	3.1452235	0.0039		-26.75
	Phenylalanine 7	3.136225	0.0039		-24.29
	S-Adenosylhomocysteine	8.3945	0.0039		-23.91
	Selenomethionine 1	2.63583075	0.0039		-34.74
	Tyrosine 4	6.9235865	0.0039		-35.95
	Tyrosine 5	3.079	0.0039		-20.60
	Tyrosine 6	3.07036725	0.0039		-22.32
	UNIDENTIFIED 7	3.181	0.0039		-22.75
	UNIDENTIFIED 8	3.1744625	0.0039		-20.60
	UNIDENTIFIED 9	3.1517965	0.0039		-26.52
	UNIDENTIFIED 10	2.3206385	0.0039		-24.36
	UNIDENTIFIED 11	2.3084785	0.0039		-26.53
	Valine 4	2.2992225	0.0039		-31.43
	UNIDENTIFIED 12	0.95341	0.004		-34.29
	Tryptophan 7	7.7455	0.0041		-22.86
	Nicotinurate 10	8.27271725	0.0042		-16.74
	Threonine 1	4.2810135	0.0043		-20.05
	Valine 5	2.26676675	0.0044		-24.26
	Phosphocreatine	3.958	0.0045		25.44
	Isoleucine 2	0.9329915	0.0048		-31.49
	O-Phosphocholine 3	3.606269	0.0048		16.80
	UNIDENTIFIED 13	4.4564145	0.0051		-27.19
	UNIDENTIFIED 14	3.967	0.0059		21.58
	Uridine 1	7.8785	0.0066		-29.06
	Myoinositol 4	3.641821	0.0067		-20.15
	UNIDENTIFIED 15	0.964489	0.007		-33.19

	Myoinositol 5	3.6147985	0.0072		-15.87
	(R)-3-Hydroxybutyric Acid 2	2.426871	0.0077		-26.07
	Beta-Alanine 2	3.1915	0.0078		-21.22
	Glucose 7	5.2418	0.0078		13.95
	Glucose 8	3.843958	0.0078		10.86
	Glucose 9	3.837573	0.0078		10.78
	Glutamate 1	2.107325	0.0078		-31.24
	Glutamate 2	2.06925	0.0078		-23.25
	Glycine	3.5715	0.0078		-15.30
	Nicotinurate 11	8.73345	0.0078		-20.45
	Norspermidine 1	1.68882425	0.0078		-27.78
	Phenylalanine 8	7.41981125	0.0078		-35.88
	Phenylalanine 9	7.3996215	0.0078		-36.29
	Phenylalanine 10	7.3839405	0.0078		-42.74
	Phenylalanine 11	7.37366075	0.0078		-34.63
	Isoleucine 3	1.02094425	0.0079		-28.43
	UNIDENTIFIED 16	0.976129	0.008		-30.51
	Isoleucine 4	1.00844425	0.0084		-28.38
	Valine 6	2.271912	0.0086		-20.44
	UNIDENTIFIED 17	2.3156385	0.0088		-22.14
	Flavin Mononucleotide	7.8095	0.0089		-35.37
	Glucose 10	3.430133	0.0091		9.80
	Inosine 4 and Hypoxanthine	8.2305	0.0097		-37.39
	Lactate 1	4.1205	0.0099		26.44
	Acetylcholine	2.142	0.0102		-30.99
	Adenosine monophosphate 1	8.598	0.0108		45.52
	Uridine 2	5.9215235	0.011		-30.78
	Glutamate 3	2.1007245	0.0113		-20.24
	Ribose	5.402	0.0113		-24.18
	(R)-3-Hydroxybutyric Acid 3	2.41629	0.0117		-24.98
	(R)-3-Hydroxybutyric Acid 4	1.2005	0.0117		-39.53
	Gamma-Glutamylcysteine 1	2.21658675	0.0117		-16.64
	Gamma-Glutamylcysteine 2	2.19575175	0.0117		-14.98
	Glucose 11	3.797585	0.0117		8.74
	Glucose 12	3.7555	0.0117		8.17
	Selenomethionine 2	2.647295	0.0117		-33.69
	Tryptophan 8	7.29929275	0.0118		-28.82
	UNIDENTIFIED 18	0.8680155	0.0118		-29.99
	Glycerophosphocholine 6	4.3325	0.0121		39.67
	Valine 7	0.9887275	0.0121		-25.56
	Adenosine	8.1415	0.0122		29.65
	Adenine	8.2165	0.0128		-33.95
	Glycerol 1	3.8075	0.0141		9.76

	Myoinositol 6	3.557273	0.0146		-11.58
	Norspermidine 2	1.67632325	0.0153		-23.13
	UMP 1	4.42397	0.0154		-26.08
	Uridine 3	5.9120235	0.0155		-24.23
	Threonine 2	4.27273825	0.0161		-17.57
	UNIDENTIFIED 19	4.4624675	0.0161		-24.73
	Myoinositol 7	3.541	0.0164		-11.55
	Inosine 5	4.4383065	0.0165		-18.87
	UDP-glucose	5.989744	0.0169		32.96
	Pi-Methylhistidine	7.1644	0.017		-23.31
	Glucose 13	3.4151865	0.018		11.87
	Uridine 4	5.9298575	0.0183		-29.03
	Methionine Sulfoxide	2.7659685	0.0184		-23.30
	UNIDENTIFIED 20	7.9168785	0.0187		-20.02
	Valine 8	1.037221	0.0187		-25.19
	Glycerol 2	3.81235	0.0188		8.99
	Threonine 3	4.254254	0.0191		-17.16
	Gamma-Glutamylcysteine 3	2.2056115	0.0195		-14.49
	Glucose 14	3.446852	0.0195		9.57
	N-Acetylglutamate 1	2.228325	0.0195		-17.97
	N-Methylhydantoin 1	4.099	0.0195		-20.63
	UDP-N-Acetylglucosamine 2	5.96753825	0.0195		32.60
	UNIDENTIFIED 21	7.573	0.0195		-18.25
	UNIDENTIFIED 22	4.48502425	0.0195		-23.62
	UNIDENTIFIED 23	4.47825525	0.0195		-22.29
	UNIDENTIFIED 24	4.36566	0.0195		-19.62
	UNIDENTIFIED 25	1.702597	0.0195		-28.60
	Uridine 5	4.3613245	0.0195		-24.69
	Uridine 6	4.2407	0.0195		-19.58
	Valine 9	0.9993275	0.0212		-22.16
	UNIDENTIFIED 26	4.41	0.0218		-22.82
	Valine 10	2.25968475	0.022		-17.86
	Adenosine monophosphate 2	6.152	0.0224		43.18
	Lactate 2	4.1421505	0.0227		11.07
	Valine 11	1.051221	0.0236		-25.81
	UNIDENTIFIED 27	4.404	0.0251		-19.36
	Myoinositol 8	3.2992265	0.0255		-21.20
	Allantoin 2	5.39	0.0256		-20.86
	7-Methyladenine 2	8.18565	0.0257		-13.35
	Choline	4.0672235	0.0261		-17.16
	Myoinositol 9	3.5485	0.0264		-10.81
	(R)-3-Hydroxybutyric Acid 5	2.4213375	0.0273		-26.68
	Glucose 15	3.74596475	0.0273		7.27

	Tyrosine 7	3.0902355	0.0273		-15.01
	UNIDENTIFIED 28	3.9405	0.0273		23.54
	Uridine 7	4.23414275	0.0273		-15.68
	Adenosine Triphosphate	8.547	0.0295		39.03
	Lactate and Gluconate	4.1321445	0.0299		18.66
	Threonine 4	4.26354675	0.0307		-16.72
	Glucose 16	3.7865425	0.0325		5.65
	Imidazole	8.32305	0.0342		14.44
	N-Methylhydantoin 2	2.929548	0.0358		-23.91
	Pantothenic acid	0.9227405	0.0366		-20.80
	Myoinositol 10	3.563	0.0372		-9.69
	Sucrose	4.0632235	0.0381		-13.70
	O-Phosphocholine 4	3.5996425	0.0388		14.67
	Glucose 17	3.7282915	0.0391		8.49
	Glucose 18	3.718924	0.0391		8.99
	Glucose 19	3.462855	0.0391		8.62
	Glucose 20	4.6792105	0.0391		7.88
	N-Acetylglutamate 2	2.23953475	0.0391		-15.84
	N-Carbamoylaspartate	2.47906925	0.0391		-27.81
	Tyrosine 8	3.2005	0.0391		-16.18
	UDP-N-Acetylglucosamine 3	5.9837385	0.0391		35.01
	UNIDENTIFIED 29	4.470371	0.0391		-23.05
	UMP 2 and Cytidine Monophosphate	8.09032775	0.0405		-49.99
	Carnosine and Histamine 2	7.149295	0.0414		-22.55
	Norspermidine 3	1.66812625	0.0417		-18.63
	Glucose 21	3.39272	0.042		6.38
	UNIDENTIFIED 30	0.911374	0.0429		-22.58
	UMP 3	4.430931	0.0433		-20.99
	Proline	3.336	0.0437		15.76
	Glucose 22	4.6503985	0.0445		8.27
	7-Methyladenine 3	4.0585	0.045		-12.64
	UDP-N-Acetylglucosamine 4	7.9454045	0.0467		22.49
	Glutamate 4	2.059403	0.0486		-15.62
	Uridine 8	7.8935	0.0492		-21.80

Table S4. *P*-values of metabolites found to be significant altered in breast muscle of birds administered 10 mg L^{-1} corticosterone (**CORT**) and 30 mg L^{-1} CORT as compared to ethanol alone as determined by a paired T-test and/or the variable importance analysis based on random variable combination analysis. Metabolite regulation is shown as a percentage of the difference between the CORT treatment vs ethanol treatment. Metabolites for which more than one nuclear magnetic resonance peak was identified as significant are represented as metabolite 1, metabolite 2, … metabolite n.

Treatment	Metabolite	Chemical shift (ppm)	T-test *P*-value	VIAVC *P*-value	Regulation
10 mg L^{-1} CORT	Glucose 1	3.90	0.001		22.54
	Glucose 2	3.84	0.001	1.61E-07	19.00
	Glycine and Glycerol 1	3.57	0.0022		28.44
	UNIDENTIFIED 1	8.26	0.0023		-69.84
	Glucose 3	3.73	0.0026		19.55
	Glucose 4	3.75	0.0042		17.89
	Glucose-6-phosphate 1 and Nicotinurate	4.00	0.0055		33.76
	Glucose-6-phosphate 2	4.02	0.0061		12.99
	Inosine 1 and Glucose-6-Phosphate 3	3.89	0.0064		43.44
	Glucose 5 and Glucose-6-Phosphate 4	3.52	0.0076		48.08
	Inosine 2 and Glucose-6-Phosphate 5	3.87	0.008		38.83
	Glucose 6 and Myoinositol 1	3.53	0.0098		53.29
	Glucose 7 and Glucose-6-Phosphate 6	5.26	0.0103		43.54
	Glucose 8 and Glucose-6-Phosphate 7 and Myoinositol 2	3.55	0.0124		38.38
	Glucose 9 and Glucose-6-Phosphate 8	3.51	0.0128		28.27
	UNIDENTIFIED 2	4.01	0.0136		21.87
	Glucose 10	3.72	0.0178		19.16
	Glucose 11 and Carnitine	3.43	0.0195		15.09
	UNIDENTIFIED 3	4.01	0.0203		33.85
	Glucose 12 and Glycerol 2	3.56	0.0204		27.44
	1,5-Anhydrosorbitol 1	3.98	0.022		30.14
	Creatine phosphate	3.03	0.0254		-16.49
	UNIDENTIFIED 4	3.96	0.0273		57.96
	1,5-Anhydrosorbitol 2	3.97	0.0283		29.97
	UNIDENTIFIED 5	3.82	0.0286		24.57
	Glucose-6-phosphate 9	3.29	0.0356		32.09
	Creatinine	4.07	0.0377		13.26
	Glucose-6-phosphate 10	5.25	0.039		28.39
	Anserine 1	2.71	0.04		-14.48
	Betaine	3.90	0.0443		18.91
	UNIDENTIFIED 6	3.86	0.0447		17.93
	N-Methylhydantoin	2.93	0.1061	1.02E-32	-25.42
	UNIDENTIFIED 7	8.57	0.1289	4.80E-07	11.44

	Glucose 13	3.71	0.2629	5.48E-08	16.68
	Glucose 14	3.83	0.3878	9.75E-20	15.37
	Glucose 15	3.69	0.4453	8.14E-21	11.71
	UNIDENTIFIED 8	3.06	0.6912	1.18E-13	2.26
	S-Adenosylhomocysteine 1	2.17	0.6916	4.16E-11	-6.19
	Anserine 2	2.67	0.8203	7.23E-13	-1.26
	S-Adenosylhomocysteine 2	2.19	0.8461	1.33E-14	-2.61
30 mg L^{-1} CORT	Glucose 1	3.43	0.00001		43.97
	Glucose 2 and Glucose-6-Phosphate 1	5.26	0.00001		77.26
	Glucose 3 and Glucose-6-Phosphate 2	3.51	0.00001		56.99
	Glucose 4 and Glucose-6-Phosphate 3 and Myoinositol 1	3.55	0.00001		72.65
	Glucose 5 and Glycerol 1	3.56	0.00001		55.13
	Glucose-6-phosphate 4	3.72	0.00001		41.01
	Histamine 1	3.03	0.00001		-28.33
	Inosine 1 and Glucose-6-Phosphate 1	3.89	0.00001		76.84
	Glucose-6-phosphate 2	4.02	0.0001		19.55
	Histamine 2	3.02	0.0001		-47.40
	Glucose-6-phosphate 3	3.29	0.0003		54.51
	Glucose 6	3.41	0.0004		29.60
	Glucose 7 and Glucose-6-Phosphate 4	5.25	0.0004		57.35
	Glucose-6-phosphate 5	4.01	0.0005		36.30
	Glucose 8	3.82	0.0006		34.76
	Glucose 9 and Glycerol 2	3.56	0.0006		42.46
	Glutathione 1	3.00	0.0006		-69.36
	Anserine 1	3.07	0.0007		-29.23
	Glucose 10	3.26	0.0007		23.34
	Anserine 2	3.09	0.001		-41.91
	Glucose 11 and Betaine 1	3.91	0.0012		33.48
	Citric acid	2.64	0.0016		-44.84
	1,5-Anhydrosorbitol 1	3.98	0.0017		45.55
	Acetic acid and Acetylphosphate	1.93	0.0022		-43.83
	Carnosine 1	8.08	0.0023		-78.86
	N-Methylhydantoin	2.93	0.0023		-61.90
	Proline 1	2.35	0.0023		-39.43
	1,5-Anhydrosorbitol 2	3.97	0.0027		42.68
	Proline 2	2.34	0.003		-44.44
	UNIDENTIFIED 2	4.01	0.003		52.87
	Glucose 12	3.90	0.0039		46.98
	Glucose 13	3.85	0.0039		22.86
	Glucose 14	3.84	0.0039		36.36
	Glucose 15	3.75	0.0039		38.47
	Glucose 16 and Glucose-6-Phosphate 6	3.52	0.0039		89.14

	Glucose 17 and Myoinositol 2	3.53	0.0039		89.57
	Glucose-6-phosphate 7	3.73	0.0039		56.38
	Glucose-6-phosphate 8 and Nicotinurate 1	4.00	0.0039		62.74
	Glycine and Glycerol 3	3.57	0.0039		61.33
	Inosine 2 and Glucose-6-Phosphate 9	3.87	0.0039		59.35
	Carnitine 1	2.44	0.0041		-54.11
	Methylmalonate 1	1.24	0.0042		-43.16
	Carnitine 2	2.43	0.0044		-62.29
	Acetylphosphate and S-Adenosylhomocysteine 1 and Glutamine 1	2.12	0.0046		-31.69
	Carnitine 3	2.47	0.0046		-55.08
	Carnitine 4	2.46	0.0047		-57.82
	Methylmalonate 2	1.25	0.0047		-41.57
	ATP 1, ADP 1, AMP 1, IMP 1	6.13	0.0048		-80.51
	Glutamine 2	2.16	0.0048		-68.52
	Niacinamide 1	8.95	0.0054		-59.53
	Glutamine 3	2.16	0.0056		-65.38
	Argininosuccinic Acid 1 and Glycylproline 1 and Ornithine 1	1.94	0.006		-95.85
	Glucose 18	3.25	0.0062		17.83
	Lactate	4.15	0.0062		11.92
	Anserine 3 and Carnosine 1	2.66	0.0068		-8.73
	Acetylcholine	2.15	0.0072		-32.75
	Niacinamide 2	8.72	0.0072		-82.11
	Tryptophan 1	7.73	0.0072		-103.36
	Niacinamide 3	7.61	0.0074		-93.38
	Uridine 1	5.91	0.0074		-118.62
	GTP or GDP 1 and Uridine 2	5.92	0.0075		-118.27
	Proline 3 and Malic acid	2.36	0.0075		-44.50
	Niacinamide 4	7.61	0.0076		-94.71
	Niacinamide 5	7.60	0.0076		-93.65
	UNIDENTIFED 3	6.02	0.0077		-96.88
	Adenine 1	8.22	0.0078		-87.13
	AMP 2 and ATP 2	8.24	0.0078		-80.34
	GTP or GDP 2 and Uridine 3	5.93	0.0078		-116.02
	Histamine 3	7.09	0.0078		-88.48
	Niacinamide 6	8.73	0.0078		-74.97
	S-Adenosylhomocysteine 2	4.46	0.0078		-47.05
	Tryptophan 2	7.56	0.0078		-109.84
	Tyrosine 1	7.21	0.0078		-76.81
	UNIDENTIFED 4	2.99	0.0078		-82.69
	UNIDENTIFIED 5	8.27	0.0081		-43.24
	Niacinamide 7	7.62	0.0084		-90.78

	Tryptophan 3	7.74	0.0088		-105.59
	4-Pyridoxic Acid	7.55	0.0091		-109.17
	Hypoxanthine	8.17	0.0091		-66.64
	Phenylalanine 1	7.45	0.0091		-97.35
	Aspartic Acid 1	2.82	0.0095		-84.27
	1,5-Anhydrosorbitol 3	3.99	0.01		35.49
	UNIDENTIFED 6	7.88	0.0101		-112.06
	AMP 3	8.60	0.0102		-94.48
	Betaine 2	3.90	0.0102		24.28
	S-Adenosylhomocysteine 3 and Proline 4	2.07	0.0102		-40.28
	N-Acetylmannosamine 1 and S-Adenosylhomocysteine 4 and Proline 5	2.06	0.0103		-48.99
	Nicotinurate 2	4.00	0.0103		35.59
	S-Adenosylhomocysteine 5 and Proline 6	2.09	0.0105		-74.65
	Tryptophan 4	7.57	0.0105		-114.96
	UNIDENTIFED 7	7.49	0.0105		-108.97
	UNIDENTIFIED 8	7.49	0.0105		-109.20
	S-Adenosylhomocysteine 6	6.10	0.0106		-39.68
	UNIDENTIFED 9	8.26	0.0107		-46.65
	UNIDENTIFED 10	6.34	0.0107		-115.62
	UNIDENTIFIED 11	8.02	0.0112		-110.98
	Carnosine 2	8.18	0.0113		-71.52
	1-Methyladenine	8.28	0.0117		-46.50
	Argininosuccinic Acid 2 and Glycylproline 2 and Ornithine 2	1.95	0.0117		-86.80
	Argininosuccinic Acid 3 and Glycylproline 3 and Ornithine 3	1.94	0.0117		-89.24
	Argininosuccinic Acid 4 and Glycylproline 4 and Ornithine 4	1.92	0.0117		-72.04
	Carnitine 5	2.48	0.0117		-62.88
	Dimethylglycine	2.94	0.0117		-49.62
	Glutamine 4 and S-Adenosylhomocysteine 7	2.14	0.0117		-63.42
	Glutamine 5 and S-Adenosylhomocysteine 8	2.13	0.0117		-63.40
	Glutathione 2	3.00	0.0117		-77.46
	Glutathione 3	2.99	0.0117		-84.29
	Glutathione 4	2.98	0.0117		-75.23
	Glutathione 5	2.95	0.0117		-76.94
	Guanidinoacetate	3.80	0.0117		20.29
	IMP 2	8.23	0.0117		-87.69
	Malonate	3.14	0.0117		-33.54
	Ornithine 5	1.91	0.0117		-70.12
	Phenylalanine 1	3.16	0.0117		-46.52
	1-Methylhistidine 1	7.01	0.0117		-108.70

	S-Adenosylhomocysteine 9	2.17	0.0117		-56.16
	Tyrosine 2	7.19	0.0117		-73.90
	Tyrosine 3	6.92	0.0117		-84.36
	Tyrosine 4	6.91	0.0117		-87.34
	UMP 1	6.01	0.0117		-111.73
	UMP 2	5.99	0.0117		-97.27
	UMP 3	5.98	0.0117		-104.68
	τ-Methylhistidine 1	3.17	0.0117		-51.86
	Glucose-6-phosphate 10	4.03	0.0118		14.69
	UNIDENTIFED 12	2.21	0.0119		-75.81
	UNIDENTIFED 13	6.03	0.0119		-89.81
	Proline 7 and Glycylproline 1	2.04	0.0121		-57.53
	UNIDENTIFIED 14	7.89	0.0122		-113.08
	S-Adenosylhomocysteine 10	4.45	0.0124		-32.49
	UNIDENTIFED 15	7.37	0.0124		-104.61
	1-Methylhistidine 2	7.70	0.0125		-110.84
	Aspartic Acid 2	2.82	0.0125		-78.08
	Aspartic Acid 3	2.80	0.0125		-66.09
	UNIDENTIFED 16	6.10	0.0125		-81.15
	UNIDENTIFIED 17	7.27	0.0126		-77.51
	S-Adenosylhomocysteine 11	6.11	0.0131		-45.45
	Valine 1	2.26	0.0135		-90.65
	Phenylalanine 2	7.33	0.0137		-77.21
	Riboflavin 1	7.38	0.0138		-92.38
	Valine 2	2.27	0.0138		-85.03
	UNIDENTIFED 18	4.67	0.0139		29.68
	Beta-Alanine 1	3.17	0.0141		-54.48
	UNIDENTIFIED 19	8.83	0.0145		-78.94
	UNIDENTIFIED 20	2.25	0.0145		-72.22
	Phenylalanine 3	7.35	0.0146		-70.65
	Aspartic Acid 4	2.79	0.0147		-62.02
	Carnosine 3	7.15	0.015		-39.52
	Valine 3	2.27	0.015		-86.26
	Valine 1	2.26	0.0151		-87.22
	UNIDENTIFED 21	8.82	0.0152		-80.15
	UNIDENTIFED 22	2.20	0.0153		-69.84
	Valine 4	2.28	0.0153		-88.46
	Riboflavin 2	7.39	0.0154		-95.96
	Valine 5	2.28	0.0154		-84.55
	O-Phosphocholine	3.24	0.0156		-14.97
	1-Methyladenosine 1	8.42	0.0158		-57.07
	Imidazole 1	7.23	0.0162		-100.91
	UNIDENTIFIED 23	3.86	0.0175		25.13

	Valine 2	2.29	0.0176		-71.13
	Glycylproline 2 and Proline 8	1.97	0.0184		-55.63
	Glycylproline 3 and Proline 9	2.01	0.0189		-56.17
	Glycylproline 4 and Proline 10	2.03	0.0191		-49.00
	Glycylproline 5 and Proline 11	2.02	0.0192		-46.46
	Mannose-6-Phosphate	5.21	0.0194		82.74
	1-Methyladenosine 2	8.15	0.0195		-74.27
	Adenine 2	8.19	0.0195		-93.21
	ADP 2	8.53	0.0195		-99.53
	Alpha-ketoisovaleric acid 1	1.15	0.0195		-87.04
	Alpha-ketoisovaleric acid 2	1.14	0.0195		-86.74
	Argininosuccinic Acid 5	1.89	0.0195		-67.89
	Argininosuccinic Acid 6 and Glycylproline 6	1.95	0.0195		-74.71
	Argininosuccinic Acid 7 and Glycylproline 7 and Ornithine 6	1.93	0.0195		-83.89
	Argininosuccinic Acid 8 and Ornithine 7	1.90	0.0195		-75.22
	ATP 3	8.53	0.0195		-84.34
	Carnitine 6	2.42	0.0195		-62.19
	Ethanol 1	1.19	0.0195		-96.84
	Ethanol 2	1.18	0.0195		-96.35
	Ethylmalonic acid	0.92	0.0195		-88.73
	Fumaric Acid	6.53	0.0195		-105.01
	Glutamine 6	2.15	0.0195		-72.33
	Glutathione 6	2.96	0.0195		-81.40
	Glutathione 7	2.94	0.0195		-87.36
	Glycylproline 8 and Proline 12	1.96	0.0195		-65.06
	GMP 1	5.95	0.0195		-109.02
	GMP 2	5.94	0.0195		-112.16
	Imidazole 2	7.29	0.0195		-89.19
	Isoleucine	0.93	0.0195		-46.58
	Pantothenic Acid	0.89	0.0195		-83.48
	Phenylalanine 4	7.44	0.0195		-71.40
	Phenylalanine 5	7.42	0.0195		-79.43
	Proline 13	3.36	0.0195		-59.94
	Proline 14	2.33	0.0195		-58.53
	S-Adenosylhomocysteine 12 and Proline 15	2.10	0.0195		-74.59
	Trimethylamine	2.90	0.0195		-102.38
	UMP 4	8.11	0.0195		-97.56
	UMP 5	8.10	0.0195		-101.07
	UNIDENTIFED 1	1.90	0.0195		-84.43
	UNIDENTIFED 24	7.50	0.0195		-108.72

	UNIDENTIFED 25	5.63	0.0195		-133.76
	UNIDENTIFIED 26	5.62	0.0195		-128.54
	UNIDENTIFED 27	2.91	0.0195		-86.08
	UNIDENTIFED 28	2.89	0.0195		-93.94
	UNIDENTIFIED 29	2.54	0.0195		-92.98
	UNIDENTIFED 30	8.21	0.0195		-101.25
	UNIDENTIFED 31	7.48	0.0195		-106.98
	UNIDENTIFIED 32	5.62	0.0195		-128.41
	UNIDENTIFED 33	5.61	0.0195		-131.71
	UNIDENTIFED 34	2.91	0.0195		-89.52
	UNIDENTIFIED 35	2.88	0.0195		-91.59
	UNIDENTIFED 36	2.24	0.0195		-67.57
	τ-Methylhistidine 2	6.99	0.0195		-83.84
	N-Acetylgalactosamine 2 and S-Adenosylhomocysteine 13 and Proline 16	2.05	0.02		-27.57
	Proline 17 and Malic acid	2.37	0.0208		-40.15
	Glucose 19 and Glucose-6-phosphate 11	3.50	0.0217		20.82
	Glycylproline 9 and Proline 18	2.00	0.0229		-55.26
	Anserine 4	2.71	0.023		-16.05
	Inosine 3	4.28	0.0257		-49.17
	UNIDENTIFED 37	9.34	0.0258		-62.58
	Inosine 4	4.44	0.0264		-39.88
	Inosine 5	4.29	0.0264		-36.25
	Beta-Alanine 2 and N-Nitrosodimethylamine and Anserine 5 and Carnosine 4	3.19	0.0266		-9.64
	S-Adenosylhomocysteine 14 and Proline 19	2.08	0.0267		-37.03
	1-Methyladenosine 3	8.46	0.0273		-62.84
	3-Hydroxyisobutyrate	1.08	0.0273		-85.52
	Adenosine Phosphosulfate	8.51	0.0273		-75.78
	Alanine 1	3.77	0.0273		-16.96
	Methionine	2.19	0.0273		-45.02
	Phenylalanine 2	3.15	0.0273		-45.74
	UNIDENTIFIED 38	7.17	0.0273		-50.92
	Glycylproline 10 and Proline 20	1.98	0.0285		-46.57
	Purine	9.11	0.0311		-57.96
	Glycylproline 11 and Proline 21	1.99	0.0316		-46.28
	S-Adenosylhomocysteine 15	8.37	0.0324		-24.85
	UNIDENTIFED 39	4.27	0.0335		-48.15
	sn-Glycero-3-phosphocholine 1	3.64	0.0365		-52.67
	UNIDENTIFED 40	1.52	0.0373		-59.10
	Inosine 6	4.28	0.039		-32.39
	3-Hydroxybutyric acid 1	1.21	0.0391		-84.07

	3-Hydroxybutyric acid 2 and Ethanol 3	1.20	0.0391		-78.79
	Lactic acid 1	1.43	0.0391		-28.25
	Lactic acid 2	1.42	0.0391		-30.27
	UNIDENTIFIED 41	3.96	0.0391		44.06
	UNIDENTIFED 42	2.23	0.0391		-77.23
	UNIDENTIFED 43	2.81	0.0391		-65.80
	Inosine 7	4.30	0.0395		-40.02
	Beta-Alanine 3	2.56	0.0397		-61.35
	Threonine	3.60	0.04		28.46
	sn-Glycero-3-phosphocholine 2	3.68	0.0426		-60.89
	Beta-Alanine 4	2.58	0.0448		-65.34
	Alanine 2	1.48	0.0455		-18.07
	Carnosine 5 and Beta-Alanine 5	3.18	0.0481		-14.24
	Glucose 1-Phosphate 1	5.47	0.1641		-94.16
	Glucose 1-Phosphate 2	5.46	0.1641		-89.79
	UNIDENTIFIED 44	8.57	0.8203		6.69
